# Search for Radiative β-Decay of the Free Neutron

**DOI:** 10.6028/jres.110.063

**Published:** 2005-08-01

**Authors:** J. Byrne, R. U. Khafizov, Yu A. Mostovoi, O. Rozhnov, V. A. Solovei, M. Beck, V. U. Kozlov, N. Severijns

**Affiliations:** Department of Physics and Astronomy, University of Sussex, Brighton BN1 9QH, U.K.; RSC Kurchatov Institute, 127562 Moscow, Russia; Petersburg Nuclear Physics Institute, 188350 Gatchina, Russia; Katholicke Universiteit Leuven, B-3001 Leuven, Belgium

**Keywords:** beta decay, cold neutrons, electroweak interactions, neutron decay, radiative corrections, radiative decay

## Abstract

Results of the first experiment to search for the radiative decay mode of the free neutron are reported. The γ-spectrum was studied in the energy region from 35 keV to 100 keV in six Cs(Tl) scintillators, each set at an angle of 35° to, and shielded from, a central plastic scintillator electron detector. Triple coincidences were recorded with recoil protons detected in a micro-channel plate. A limit for the branching ratio BR < 6.9 × 10^−3^ (90 % confidence level) was obtained, which is greater that the theoretical prediction by not more than a few tenths of a percent.

## 1. Introduction

The emission of a weak continuous γ-ray spectrum during the *β*-decay of the free neutron

$$n\Rightarrow p+e^{-}+{\bar{\nu }}_{e}+\gamma$$ (1)

provides an example of a general process described as internal bremsstrahlung, which results from the displacement of electric charge which occurs whenever charged particles are created or destroyed. Since the γ-ray has spin *J_γ_* = 1, photon emission in the dipole mode satisfies fully the conditions of angular momentum conservation which apply to the dominant radiationless branch. However the violation of parity equires that the photons be emitted as an incoherent mixture of E1 and M1 radiations and therefore the radiation is left circularly polarized and the transfer of helicity from electron to photon approaches 100 % at the end-point of the spectrum.

**Fig. 1:**
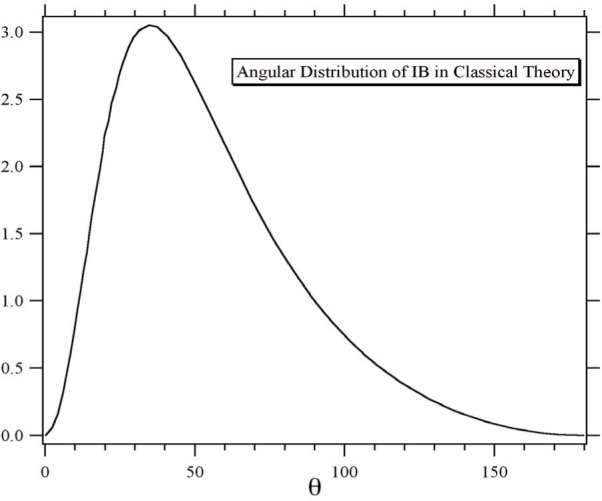
Angular distribution of inner bremsstrahlung from neutron *β*-decay in the classical approximation [1].

The main features of radiative *β*-decay have been derived in the classical approximation by Jackson [1] who assumes that an electron is created at the origin at *t* = 0 with constant velocity ***v*** = *cβ*, in which case radiation of angular frequency *ω* is emitted in the direction of the unit vector ***n*** with an angular distribution in energy per unit time per unit interval of angular frequency

$$\frac{dI\left(\omega \right)}{d\Omega }=\frac{e^{2}\beta_{e}^{2}}{4\pi^{2}c}\frac{\sin^{2}\theta }{{\left(1-\beta_{e}\cos\theta \right)}^{2}},$$ (2)

where *β*_e_ cos *θ* = ***n*** · *β*_e_. The factor sin^2^ *θ* is characteristic of dipole radiation and the denominator (1−*β*_e_ cos *θ*)^2^ arises as a consequence of retardation. Thus, for a value of *β*_e_ = 0.82, which is about average for neutron *β*-decay, the radiation pattern peaks at a value of θ≃35°. This is shown in Fig. 1.

Integrating over the angles and letting *N*(*E*_γ_) represent the number of photons emitted per unit energy interval where *N*(*ħω*)(*ħω*)d(*ħω*) = *I*(*ω*)d*ω*, then we find that

$$N\left(E_{\gamma }\right)=\frac{\alpha }{\pi }\left(\frac{1}{E_{\gamma }}\right)\left\{\frac{1}{\beta_{e}}\ln\left[\frac{1+\beta_{e}}{1-\beta_{e}}\right]-2\right\}.$$ (3)

Since the maximum energy radiated cannot exceed (*E*_0_ − *E*_e_), where the end-point energy $E_{0}≃\Delta =\left(m_{n}–m_{p}\right)c^{2}$, it follows that the total number of photons radiated is given by the formula

$$\begin{array}{l} N=\int_{0}^{\Delta -E_{å}}N\left(E_{\gamma }\right)dE_{\gamma }=\\ \;=\frac{\alpha }{\pi }\langle \frac{1}{\beta_{e}}\ln\left[\frac{1+\beta_{e}}{1-\beta_{e}}\right]-2\rangle \int_{0}^{\Delta -E_{e}}dE_{\gamma }/E_{\gamma }. \end{array}$$ (4)

The integral in Eq. (4) diverges logarithmically as *E_γ_* ⇒ 0, a result which is described as the infra-red divergence. In this case and in general, the combination of the divergences arising from the emission of real soft photons and the exchange of virtual soft photons cancel completely for all radiative processes and to all orders of *α*. Thus, in the case of radiative neutron *β*-decay, the number of photons recorded per decay depends on the lowest photon energy *E_γ_*, _min_ which can be observed above noise in the detector. In other respects the distribution in energy and angle is determined almost entirely by the kinematical constraints.

The electrons from neutron decay can also create external bremsstrahlung by collisions with atomic nuclei which is forwarded directed. The cross-section for this process may be evaluated from the Bethe-Heitler formula [2] and the evident similarity between the internal and external bremsstrahlung distributions in energy is sufficient indication that to distinguish these components in the total γ-ray spectrum presents a formidable experimental problem.

## 2. The Outer Radiative Correction

### 2.1 Decay Rate of the Free Neutron

The lifetime *τ*_n_ for the free neutron decay rate is given by the formula

$$\tau_{n}^{-1}=f\left(1+\delta_{R}\right)\left(\ln2\right){\left({G^{\prime }}_{V}\right)}^{2}\left[1+3\lambda^{2}\right]/K,$$ (5)

where *K* / =(*ħc*)^6^ = 8.120270 ±0.000010 ×10^−7^GeV^−4^s and the vector coupling constant ${G^{\prime }}_{V}$ is expressed in terms of the Fermi coupling constant *G*_F_ extracted from muon decay by the relationship ${\left({G^{\prime }}_{V}\;\right)}^{2}=V_{\text{ud}}^{2}{\left(G_{F}\right)}^{2}\left(1+\Delta_{R}^{V}\right)$, and $\Delta_{R}^{V}$ is a nucleus-independent inner radiative correction. The outer radiative correction is contained in the correction factor (1 + *δ*_R_) to the integrated statistical factor *f*

$$\begin{array}{l} f\left(1+\delta_{R}\right)=\int_{m_{e}c^{2}}^{E_{0}}dE_{e}\rho \left(E_{0},E_{e}\right)F_{C}\left(1,E_{e}\right)\\ \left[1+R\left(E_{e}\right)\right]\left\{1+r\left(E_{e}\right)\right\}/{\left(m_{e}c^{2}\right)}^{5}, \end{array}$$ (6)

where *ρ* (*E*_0_, *E*_e_) = |*p*_e_|*E*_e_(*E*_0_ − *E*_e_)^2^ is the phase space factor, *R* (*E*_e_) is a recoil order correction and *r* (*E*_e_), the outer radiative correction, is expressed in terms of the universal Sirlin Function *g*(*β*_e_) [3]

$$r\left(E_{e}\right)=\left(\frac{\alpha }{2\pi }\right)g\left(\beta_{e}\right).$$ (7)

The inner correction has been evaluated by Wilkinson [4] and more recently by Towner and Hardy [5] who arrive at a value *f* (1+*δ*_R_) = 1.71489 ± 0.00002.

### 2.2 Radiative Free Neutron Decay

The branching ratio for this process has been explicitly evaluated by Gaponov and Khafizov [6], who derive a differential cross-section for the radiative decay branch of the form

$$\frac{d^{2}\Gamma }{dE_{e}dE_{\gamma }}=\left(\frac{\alpha }{2\pi }\right)\left[1+3\lambda^{2}\right]\left\{\Psi \left(E_{e},E_{\gamma }\right)+\alpha_{0}\Phi \left(E_{e},E_{\gamma }\right)\right\},$$ (8)

where the first term is proportional to the normal neutron decay rate, while the second term depends on the electron-antineutrino angular correlation coefficient *a*_0_ = [1 − *λ*^2^]/[1+3*λ*^2^]. There are no terms in *λ* consistent with the fact that, in the absence of helicity measurements, the absolute radiative decay rate is parity conserving. Following an integration over the angles, the terms proportional to *a*_0_ are of recoil order, and are omitted together with Coulomb corrections. The resultant single differential cross section then reduces to the form

$$\begin{array}{l} \frac{dT}{dE_{e}}=\int_{E_{\gamma ,\min}}^{\Delta -E_{e}}dE_{\gamma }\frac{d^{2}\Gamma }{dE_{e}dE_{\gamma }}=\left(\frac{\alpha }{2\pi }\right)\tau_{n}^{-1}\\ \left[\frac{\rho \left(E_{0},E_{e}\right)}{\int_{m_{e}c^{2}}^{E_{0}}dE_{e}\rho \left(E_{0},E_{e}\right)}\right]\left(g_{e}\left(\beta_{e}\right)+g_{p}\left(\beta_{e}\right)\right), \end{array}$$ (9)

where the functions *g*_e_*(β*_e_, *E_γ_*,_min_) and *g*_p_(*β*_e_, *E_γ_*,_min_) describe the electron and proton contributions to the radiative branch respectively for *E_γ_* > *E_γ_*,_min_. Their sum coincides with the Sirlin function *g*(*β*_e_) excluding the contribution from soft virtual processes.

**Fig. 2:**
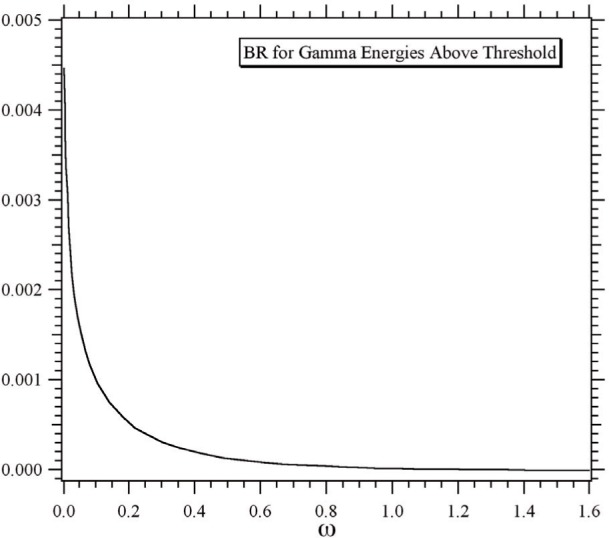
Computed branching ratio (BR) for the radiative *β*-decay for *γ* rays of energy *E_γ_* > *E_γ_*,_min_ = *ħω.*

**Fig. 3:**
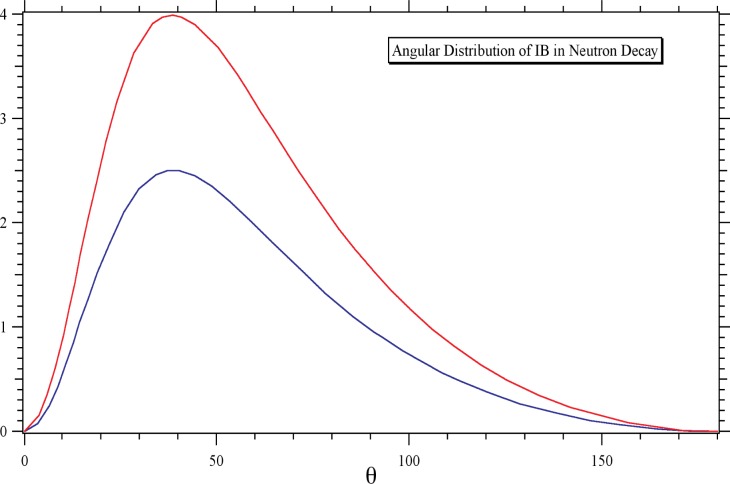
Angular distribution of inner bremsstrahlung from neutron *β*-decay for *E_γ_*_, min_ = 25 keV (upper curve) and 50 keV (lower curve).

Some results of the theoretical study are displayed in Figs. 2 and 3. Figure 2 illustrates the calculated branching ratio as a function of *E_γ_*,_min_ expressed in units of the electron mass. This leads to a value for the energy range 35 keV < *E_γ_* < 100 keV of about 0.1 %. Figure 3 shows the angular distribution of the radiation for values of *E_γ_*,_min_ of 25 keV and 50 keV. The striking similarity between Fig. 3 and the classical result in Fig. 1 is significant.

## 3. Experimental Method

**Fig. 4:**
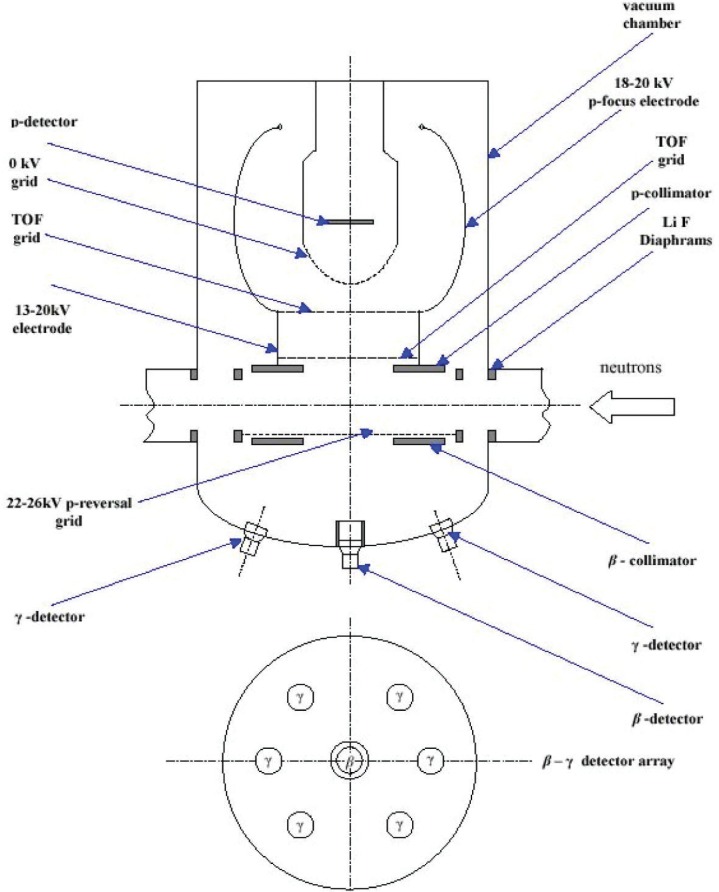
Experimental apparatus used in search for radiative neutron *β*-decay.

The experimental setup is shown in Fig. 4. The cold neutron beam with an intensity of approximately 10^11^ s^−1^ to 10^12^ s^−1^ exits from a guide containing a collimator system made from LiF diaphragms spaced 1 m apart. The decay volume is sampled by three detectors: a micro-channel plate (MCP) proton detector; a 3 mm thick, 7 cm diameter plastic scintillator electron detector, and a set of six *γ*-ray detectors, each composed of a 5 mm thick, 7 cm diameter Cs(Tl) scintillators mounted on a photo-tube. To ensure the maximum efficiency for detecting the radiative decay events the *γ*-ray detectors are each set at an angle of 35° to the electron detector (Figs. 1 and 3) and are isolated from it by 6 mm thick lead shields. By recording coincidences between *γ*-ray and electron events the external bremsstrahlung, which is generated mainly in the electron detector, can in principle be suppressed. However in order to reduce the contribution of random double coincidences, it is also necessary to observe a triple coincidence with the recoil proton.

Recoil protons pass from the decay zone through a cylindrical time-of-flight electrode and are focused onto the MCP by spherical focusing electrodes. These are each set at about 18 kV to 20 kV and the combined system is cylindrically symmetric about an axis normal to the MCP. The focusing action is brought about by a pair of grids which separate the time-of-flight and focusing electrodes each of which is set at about 18 kV to 20 kV. The MCP is isolated by a third grid maintained at ground potential. In order to collect those protons which take off in a direction away from the MCP, a fourth deflecting grid set at 22 kV to 26 kV is inserted between the decay volume and the electron detector to bring about a 4π collection solid angle for protons. Unfortunately nothing approaching this degree of collection efficiency was attained due to the presence of a 7 cm diameter plastic collimator which was incorrectly positioned between the decay region and the proton detector system.

An important role was played by the system of LiF diaphragms which suppressed the *γ*-ray background rate to about 100 s^−1^ and about the same level of background was observed in the electron detector. It is likely that these events were associated with neutron decay since they disappeared immediately the beam was switched off. The main problem encountered in the experiment arose in connection with the proton detector which turned out to be very sensitive to the vacuum conditions in the experimental chamber. In the event an unexpected contamination of the chamber with oil vapours prevented the establishment of a good vacuum for most of the running time. This caused a large background in the proton detector and led to large number of false triple coincidences. The maximum number of genuine electron-proton coincidences never amounted to more than a few per minute. This was due to the low efficiency of the MCP which, even after the vacuum problem had been solved, could not be replaced during the running time.

## 4. Experimental Results

**Fig. 5:**
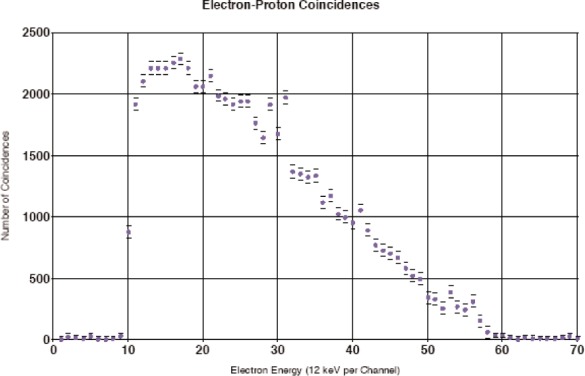
Spectrum of *β*-particles in coincidence with recoil protons.

**Fig. 6:**
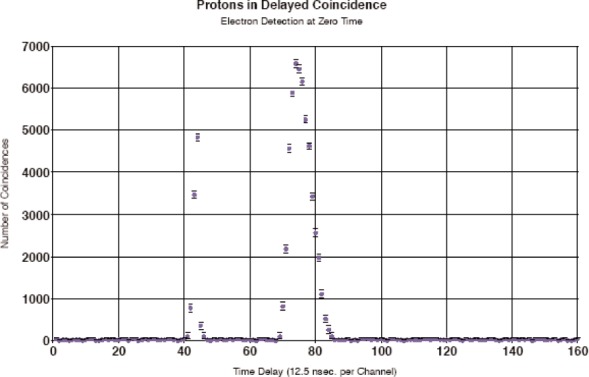
Timing spectrum of recoil protons in delayed coincidence with *β*-particles.

The first test experiments were carried out on the cold neutron beam PF1B at the Institut Laue-Langevin in Grenoble, France in April/May 2002 with the aim of testing the experimental setup, and some of the results are illustrated in Figs. 5 and 6 [7]. Figure 5 shows the spectrum of electrons observed in coincidence with protons. The coincidence counting rate for these double coincidences came to about 0.5 s^−1^ to 1 s^−1^ corresponding to a background suppression factor of about 10^3^. Figure 6 shows the timing spectrum of protons in delayed coincidence with electrons where each channel corresponds to 12.5 ns. The center point of the peak located near channel 44 corresponds to the zero of time for the detection of protons in delayed coincidence with electrons and this peak contains prompt coincidences between backward scattered external bremsstrahlung quanta generated by electrons which penetrate into the electron detector which are registered in the proton detector. The second peak centred near channel 74 derives from genuine electron-proton coincidences.

A total of eight events were recorded in the timing spectrum of genuine proton events detected in triple coincidence with electrons and gamma-rays in the energy range 35 keV to 100 keV.

From these data an upper limit for the branching ratio (BR) for the radiative decay branch can be estimated. Adopting the procedure recommended by the Particle Data Group [8] to deduce upper limits for Poisson processes when only a small number of events has been observed, this may be expressed in terms of the triple coincidence rate *N*_T_ and the electron-proton coincidence rate *N*_D_,

$$BR\leq k\left(\frac{N_{T}}{N_{D}}\right){\left(\varepsilon_{\gamma }\Omega_{\gamma }f\right)}^{-1},$$ (10)

where *ε_γ_* and *Ω_γ_* represent the efficiency and solid angle respectively for detection of *γ*-rays and *Ω_γ_f* represents the integral of the normalized *γ*-ray distribution function *f* over the steriometric angle of the six *γ*-ray detectors. The result of Eq. (10) does not depend on the efficiency or solid angle of the electron and proton detectors. For an estimated background count of 1.5 in the region where the triple coincidence peak is expected and *N*_T_ = 1, *N*_D_ = 5382, *ε_γ_* = 1 and *Ω_γ_f* = 0.084, the recommended procedure arrives at a value *k* = 3.11, corresponding to an upper limit for the branching ratio between 35 keV and 100 keV of 6.9 × 10^−3^ (90 % confidence level).

Since carrying out the experiments described above the apparatus has been rebuilt producing a vacuum of 10^−6^ mbar, and a new MCP has been installed. With an improved geometry approaching 4π solid angle for proton detection we expect to improve the observed rate of electron-proton coincidence detection by two orders of magnitude [9].
